# The importance of information on relatives for the prediction of genomic breeding values and the implications for the makeup of reference data sets in livestock breeding schemes

**DOI:** 10.1186/1297-9686-44-4

**Published:** 2012-02-09

**Authors:** Samuel A Clark, John M Hickey, Hans D Daetwyler, Julius HJ van der Werf

**Affiliations:** 1 University of New England, Armidale, NSW 2351, Australia; 2 CRC for Sheep Industry Innovation, University of New England, Armidale, NSW 2351, Australia; 3 Biosciences Research Division, Department of Primary Industries, 1 Park Drive, Bundoora, Vic. 3083, Australia

## Abstract

**Background:**

The theory of genomic selection is based on the prediction of the effects of genetic markers in linkage disequilibrium with quantitative trait loci. However, genomic selection also relies on relationships between individuals to accurately predict genetic value. This study aimed to examine the importance of information on relatives versus that of unrelated or more distantly related individuals on the estimation of genomic breeding values.

**Methods:**

Simulated and real data were used to examine the effects of various degrees of relationship on the accuracy of genomic selection. Genomic Best Linear Unbiased Prediction (gBLUP) was compared to two pedigree based BLUP methods, one with a shallow one generation pedigree and the other with a deep ten generation pedigree. The accuracy of estimated breeding values for different groups of selection candidates that had varying degrees of relationships to a reference data set of 1750 animals was investigated.

**Results:**

The gBLUP method predicted breeding values more accurately than BLUP. The most accurate breeding values were estimated using gBLUP for closely related animals. Similarly, the pedigree based BLUP methods were also accurate for closely related animals, however when the pedigree based BLUP methods were used to predict unrelated animals, the accuracy was close to zero. In contrast, gBLUP breeding values, for animals that had no pedigree relationship with animals in the reference data set, allowed substantial accuracy.

**Conclusions:**

An animal's relationship to the reference data set is an important factor for the accuracy of genomic predictions. Animals that share a close relationship to the reference data set had the highest accuracy from genomic predictions. However a baseline accuracy that is driven by the reference data set size and the overall population effective population size enables gBLUP to estimate a breeding value for unrelated animals within a population (breed), using information previously ignored by pedigree based BLUP methods.

## Introduction

Genomic selection (GS) is a method that uses genomic information to estimate breeding values and rank selection candidates in livestock breeding programs. It has become widely used in some livestock industries e.g. dairy cattle and pig improvement programs. Initial studies on genomic evaluation have suggested that GS predicts the effects of markers in linkage disequilibrium (LD) with quantitative trait loci (QTL). This implies that accurate predictions of breeding value may persist for several generations, allowing for: 1) a reduced number of phenotypic measurements in each generation [1] and; 2) the possibility of accurate predictions across different breeds provided sufficient marker density [2]. Habier et al. [3] proposed that genomic predictions also rely on the genetic relationships between individuals with phenotypic records, usually known as the reference data set, and those whose breeding value is to be predicted [4,5]. The following question arises: does an animal that has its breeding value predicted from genomic information require relatives in a reference data set?

The reference data set is used to gain information on important phenotypes and genotypes so that genomic estimated breeding values (GEBV) can be highly accurate for selection candidates. The makeup and size of this data set, combined with the methods used to predict the breeding value, govern the accuracy achieved in many breeding schemes [6]. Due to the cost of measuring genotypes and phenotypes on large numbers of individuals, it has been suggested that using a specially selected reference data set may be a cost effective way of gaining the economic advantage presented by genomic selection, especially for species such as beef cattle and sheep that do not have the nucleus structure of the dairy cattle, pig and poultry industries [7,8].

Various methods are used to predict breeding values from genomic data. These range from variable selection methods such as BayesB, which allows only a small number of loci to have an effect, some of them potentially large, to gBLUP, which assumes equal variance across all loci [9]. Empirical evidence across livestock populations has shown that in many cases these methods obtain very similar accuracies of the estimated breeding value [10]. This suggests that additive genetic variation for many traits is controlled by many genes with a small effect, somewhat like Fisher's (1918) [11] 'infinitesimal model'.

The gBLUP method to estimate genomic breeding values has been widely described [5,10,12,13]. This method uses genomic information in the form of a genomic relationship matrix (GRM) that defines the additive genetic covariance between animals [14]. The GRM then replaces the pedigree-based numerator relationship matrix (NRM) in the traditional BLUP equations. The GRM is expected to give a more accurate estimate of the covariance between individuals, however, it is important to understand how much accuracy is gained from improved measures of covariance among known relatives and how much is gained from information on distant 'relatives' previously ignored via the pedigree method.

Goddard [6] and Daetwyler et al. [15] presented methods to predict the accuracy of genomic selection. These methods predict accuracy based on the effective population size, the number of animals in the reference data set, the heritability and the effective number of chromosome segments segregating within the population. Hayes et al. [5] extended the approach by Goddard [6] to consider information from relatives and illustrated that the GRM uses information on true relationships, which can vary around the expected additive genetic relationship predicted from pedigree. This study will compare the accuracy of an animal's breeding value that has a strong pedigree relationship with a reference data set with that of an animal that is essentially unrelated to the reference data set, and discuss the effect of these relationships on the design of reference data sets used in genomic selection breeding schemes.

## Methods

To examine the effect of relationships between animals in the test and reference data sets, we used both computer simulation and real phenotypic data from the Australian Sheep CRC Information Nucleus Flock (INF). The INF animals are located at eight sites across Australia and managed by CRC partner organizations, including The University of New England, NSW Department of Primary Industries, Victorian Department of Primary Industries, South Australian Research and Development Institute and The Department of Agriculture of Western Australia. The experimental data in this paper were obtained according to protocols approved by the Animal Ethics committees of these organizations.

### Simulated Data

Genotype simulations were conducted using the Markovian Coalescence Simulator (MaCS) [16] to simulate 2 000 base haplotypes, with an effective population size (*N_e_*) of 100. As described in Clark et al. [17], thirty chromosomes each with base haplotypes of a 100 cM region (1·10^8 ^base pairs) were simulated, with a per site mutation rate of 2.5·10^-8^. The total number of SNP segregating on the genome was approximately 1 670 000 (SNP sequence). Sixty thousand SNP markers and 10 000 QTL were randomly selected from the SNP sequence in the base generation to be used in the genomic analysis (9428 QTL segregating in the final generation). Therefore each SNP had a 3% chance of being used as a marker and a 0.5% chance of being used as a QTL. The additive effect of each QTL was drawn from a gamma distribution with a shape and scale of 0.4 and 1.66 respectively [8] and had a 50% chance of being positive or negative.

The base population haplotypes were randomly allocated to 80 base males and 2 000 base female animals of a simulated population structure, with 10 subsequent generations receiving these haplotypes via mendelian inheritance, allowing recombination to occur according to genetic distance, i.e. 1% recombination per cM. The population was simulated for 10 generations and each generation contained 4000 animals, half male and half female. Eighty males were randomly selected in each generation and each male was randomly mated to 25 females, which each had two offspring per generation. Only breeding animals were allocated breeding values and phenotypes.

The true breeding value (*TBV*) of each animal was determined using:

$$TBV_{k}=\overset{nr\;of.QTL}{\underset{j=1}{\sum }}\beta_{j}\cdot Q_{kj}$$

where *β_j _*is the additive effect of QTL genotype (*j*) and *Q_kj _*is the QTL genotype at locus *j *which is coded as 0, 1, or 2 and is the number of copies of the QTL that an individual (*k*) carries. Trait phenotypes were simulated based on a heritability (*h*^2^) of 0.3 and all other variation in phenotype was due to a random environmental effect drawn from a normal distribution with variance $\sigma_{e}^{2}$.

Three reference data sets of 1 750 animals were formed for the simulation study. Reference data set 1 (closely related) consisted of animals from the 10^th ^generation. This reference data set was constructed such that animals in the test data set had 20 half sibs in the reference data set. Reference data set 2 (distantly related) also consisted of animals in the 10^th ^generation but there were no close relationships between animals in the test and reference data sets. However there were some second degree relationships (1^st ^cousins) between the two data sets. The final reference data set (unrelated) consisted of females from generation 1 and resulted in a very low or zero relationship between the two data sets. The accuracy of prediction was assessed in the test data set which consisted of 250 animals from the 10^th ^generation and the average correlation between TBV and estimated breeding value (genomic or pedigree based) was calculated over 10 replicates of the simulation study.

### Data analysis

As in Hayes et al. [13], we assumed a model

$$y=1_{n}\mu +Zg+e$$

where **y **is a vector of phenotypes, μ is the mean, 1_n _is a vector of 1s, **Z **is a design matrix allocating records to breeding values, g is a vector of breeding values for animals in the reference set and the test set and e is a vector of normal deviates with variance $\sigma_{e}^{2}$. Furthermore $\text{v}\left(\text{g}\right)=G\sigma_{g}^{2}$ where **G **is the genomic relationship matrix (GRM), and $\sigma_{g}^{2}$ is the genetic variance for this model. The GRM (**G**) was formed using the method as defined by VanRaden [18]

Traditional best linear unbiased prediction (BLUP) was also performed, using a deep (BLUP-D), 10 generation pedigree or a shallow, single generation pedigree (BLUP-S). Traditional BLUP ignores genomic data and relies on information from ancestors using a numerator relationship matrix (**A**) based on pedigree information. This method uses the same model as gBLUP (above) however with the vector of additive genetic values g replaced by a, with $\text{v}\left(\text{a}\right)=A\sigma_{a}^{2}$ where **A **is the numerator relationship matrix and $\sigma_{a}^{2}$ is the additive genetic variance. Variance components for both BLUP methods were estimated with ASREML [19] and the model solutions yielded estimated breeding values.

### Merino sheep phenotypic data

The reference data set consisted of phenotypic and genotypic records for the Merino sheep from the Australian Sheep Cooperative Research Centre information nucleus flock (INF) [20]. The traits ultrasound scanned eye muscle depth (EMD; 1781 animals) and live weight at ultrasound scanning (SC_WT; 1743 animals) were evaluated. Scanned EMD is used to estimate the size of the rib-eye muscle, which produces high value cuts of meat, and SC_WT is highly correlated to an animal's weight at post weaning. Animals in the INF were sired by rams from the wider Merino population; these sires were chosen to maximize the connectedness with the Australian sheep flock by sampling artificial insemination sires from a wide range of sheep breeders.

The test data set consisted of a population of Australian Merino industry sires with highly accurate Australian sheep breeding values (ASBV). Information about ASBV definitions can be found at the following website maintained by Australian Wool Innovation Ltd and Meat and Livestock Australia [21]. The industry sires were divided into closely, distantly and unrelated groups based on their pedigree relationship the animals in the INF flock. The maximum relationship of an animal in the test data set with an animal in the reference data set ranged from 0.125 to 0.5 (no progeny included) for the 48 closely related test animals, from 0 to 0.125 for the 60 distantly related test animals, and the 53 unrelated test animals shared no pedigree relationship to the reference data set.

### Genotypic data

All animals were genotyped using the Illumina 50 K ovine SNP chip (Illumina Inc., San Diego, CA, USA), which includes 54 977 SNP. Following the genotyping procedures, quality control measures were applied to all SNP as follows: SNP were removed if they had a call rate of less than 95%, a GC score (proportion of guanine-cytosine pairs) of less than 0.6, a minor allele frequency of less than 0.01, a SNP heterozygosity of greater than 3 s.d. from the mean (mean heterozygosity, 0.374; s.d., 0.129), were not in Hardy-Weinberg equilibrium (a P-value cut-off of 1·10^-15^), had no genome location or were in greater than 0.99 LD with another SNP on the chip [20]. After these quality control measures were applied, 48 640 SNP were used. Missing genotypes were imputed using fast PHASE [22].

### Data analysis

The following fixed effects were fitted in both trait models: sex, birth type, rearing type, age of dam, contemporary group (birth year • birth month • site • management group) and age-at-trait recording, SC_WT was fitted in the analysis of EMD.

As in Daetwyler et al. [20], the following model was assumed

y=Xb+Zg+e

where **X **is a design matrix relating the fixed effects (as described above) to each animal and **b **is a vector of fixed effects. Genetic evaluation was undertaken using BLUP-D, using a deep pedigree of 7277 animals that ranged from one to eight generations in length (depending on the individual), and gBLUP as defined above. Variance components for all methods were again estimated using ASREML [19] and the model solutions yielded estimated breeding values.

### Validation and accuracy

The empirical accuracy (r_(cor)_) for the Merino data analysis was evaluated as the Pearson product-moment correlation between the GEBV and a progeny test ASBV of the animals in the test data set. The empirical accuracy may be an underestimate of the 'real' accuracy because the ASBV accuracies are below 1. The validation sires had an ASBV accuracy greater than 0.5 and the mean accuracy was 0.85 for EMD and 0.9 for SC_WT. The ASBVs used were calculated such that they included no information from animals in the reference data set. The Merino sheep population is highly heterogeneous and can be divided into strains defined as fine, medium and strong wool types. Correlations between GEBV and ASBV were calculated after accounting for the effect of strain.

The empirical accuracy of the breeding values estimated in the test set, for the simulation example was defined as the correlation between the true and estimated breeding value. The accuracy was also estimated for each individual as: $r_{\left(PEV\right)}=\sqrt{(1-\left(PEV/G_{ii}\sigma_{a}^{2}\right)}$ where; *PEV *is the prediction error variance estimated using elements from the mixed model equations, ***G**_ii _*is the diagonal of the GRM for animal *i *and is substituted for ***A**_ii _*in traditional BLUP, $\sigma_{a}^{2}$ is the additive genetic variance. Furthermore, $PEV=C_{ii}\sigma_{e}^{2}$ where; ***C**_ii _*is the diagonal of inverse of the coefficient matrix for animal *i *and $\sigma_{e}^{2}$ is the residual variance (See Appendix 1).

To determine the effect of an individual's relationship to the reference data set on the accuracy of genomic predictions, a range of comparisons were made between varying definitions of relatedness and an individual's GEBV accuracy using r_(PEV)_. Four measures of genomic relatedness were considered: a) An animal's mean relationship with the reference data set; b) its maximum relationship; c) its mean top 10 relationships and d) its mean top 100 relationships.

## Results

### Simulation

Breeding values that were estimated using gBLUP always achieved a higher accuracy than both pedigree-based BLUP methods. When animals in the test and reference data sets were closely related (reference data set 1), all methods gave an accurate prediction of breeding value (Table 1). When the two data sets were distantly related (reference data set 2), accuracies were generally lower but the reduction in accuracy was much smaller for gBLUP than for the pedigree-based BLUP methods. Furthermore, when the two data sets' were unrelated (reference data set 3), gBLUP gave much higher accuracies than both BLUP methods.

**Table 1:** Empirical accuracy^1 ^(± S.E.)^2 ^using genomic and pedigree based methods in simulated data.

Method	Relationship to reference [pedigree relationship]		
	
	Close [0.25]	Distant [0.125]	Unrelated[0.003]
BLUP-S	0.39 (0.021)	0.00 (0.000)	0.00 (0.000)
BLUP-D	0.42 (0.019)	0.21 (0.031)	0.03 (0.016)
gBLUP	0.57 (0.014)	0.41 (0.034)	0.34 (0.021)

^1 ^Calculated as the correlation between estimated and true breeding values for 250 animals with no phenotype. Breeding values estimated using genomic (gBLUP) and pedigree (BLUP-S and BLUP-D) based methods in simulated data for groups of animals with different relationships to the reference data set.

^2 ^Standard error of means of 10 replicates

There was no significant difference in accuracy between BLUP-S and BLUP-D when the animals in the test and reference data sets had a close relationship. However, when a shallow pedigree was used, and animals in the test and reference data sets were distantly related or unrelated, all breeding values estimated using BLUP-S were zero. In contrast, BLUP-D predicted a breeding value with a significant accuracy when the reference and test data sets shared a distant relationship and accuracy reduced to close to zero when animals in the reference and test data sets were unrelated.

**Figure 1:**
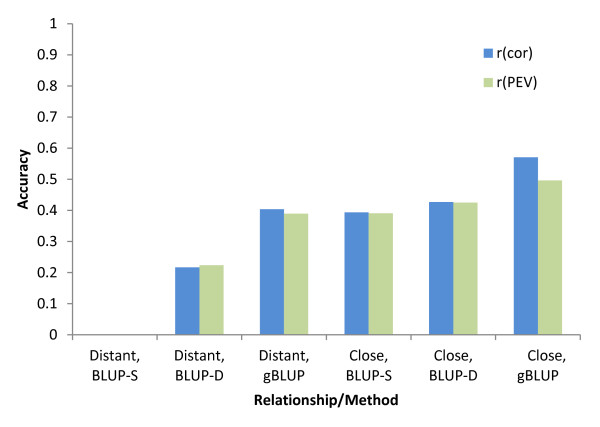
**Estimates of accuracy based on the PEV from the coefficient matrix (r_(PEV)_) and based on the correlation between estimated and true breeding values (r_(cor)_) for the close and distantly related individuals using genomic (gBLUP) and pedigree (BLUP-S and BLUP-D) based prediction methods**.

The estimate of accuracy, r_*(*PEV*)*_, when averaged over the test data set, was similar to the empirical accuracy of the group r_(cor)_. The largest difference between the two accuracy estimates was observed for gBLUP, where r_*(*PEV*) *_under-estimated the realized accuracy when half-sib family information was used (Figure 1).

### Merino sheep data analysis

There was no significant difference in the empirical accuracy of the estimated breeding values between pedigree and genomic methods for EMD for close relationships (Table 2). As in the simulated data, when the relationship between the test and reference data sets was reduced the difference in accuracy between gBLUP and BLUP-D increased. In addition, there was still a significant amount of accuracy achieved when using gBLUP in unrelated animals.

**Table 2:** Empirical^1 ^(r_(cor)_) and estimated accuracy^2 ^(r_(pev)_) using genomic and pedigree based methods for the Merino EMD data.

	Accuracy					
	
Method	Relationship to reference [Maximum pedigree relationship]					
	**Close [0.5]**		**Distant [0.125]**		**Unrelated [0.00]**	
	r_(cor)_	r_(pev)_	r_(cor)_	r_(pev)_	r_(cor)_	r_(pev)_
BLUP-D	0.46	0.21	0.17	0.07	0	0
gBLUP	0.43	0.5	0.29	0.31	0.28	0.27

^1^Correlation between breeding values estimated based on genotype and based on a progeny test. Breeding values estimated using genomic (gBLUP) and pedigree (BLUP-S and BLUP-D) based methods in the Merino EMD data for groups of animals with different relationships to the reference population.

^2 ^Derived from the mixed model equations

Results for SC_WT are given in Table 3 showing that gBLUP gave higher accuracies than BLUP-D. When the relationship between the test and reference data sets was reduced, gBLUP was again considerably more accurate than BLUP-D.

**Table 3:** Empirical^1 ^(r_(cor)_) and estimated accuracy^2 ^(r_(pev)_) using genomic and pedigree based methods for the Merino SC_WT data.

	Accuracy					
**Method**	**Relationship to reference [Maximum pedigree relationship]**					
	
	**Close [0.5]**		**Distant [0.125]**		**Unrelated [0.00]**	
	
	r_(cor)_	r_(pev)_	r_(cor)_	r_(pev)_	r_(cor)_	r_(pev)_

BLUP-D	0.15	0.43	0.21	0.05	0	0
gBLUP	0.27	0.57	0.24	0.29	0.18	0.27

^1 ^Correlation between breeding values estimated based on genotype and based on a progeny test. Breeding values estimated using genomic (gBLUP) and pedigree (BLUP-S and BLUP-D) based methods in the Merino SC_WT data for groups of animals with different relationships to the reference data set.

^2 ^Derived from the mixed model equations

For the EMD example, the estimated and empirical accuracies were very similar when using gBLUP. However for the SC_WT scenarios, there was a large difference between the estimated accuracy and the empirical accuracy.

**Figure 2:**
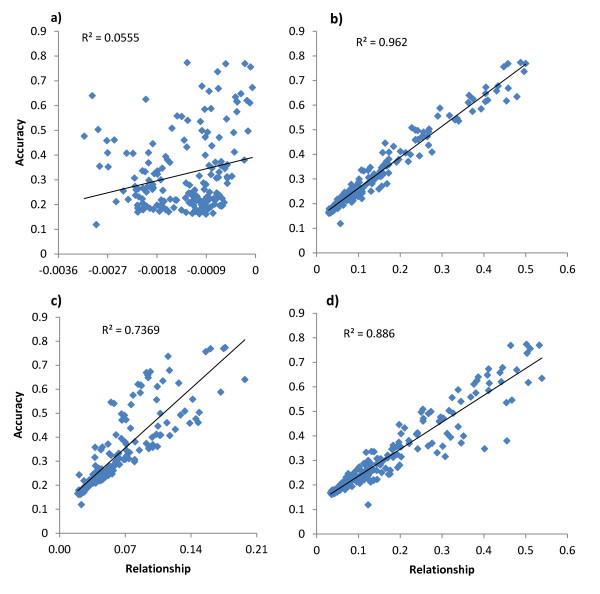
**Estimates of accuracy predicted using gBLUP and plotted against different measures of relationship between an animal in the test data set with animals in the reference data set**. These measures include: a) The mean relationship, b) The average of the top ten relationships, c) The average of the top 100 relationships and d) The maximum relationship to the reference population.

To predict the accuracy of a GEBV based on an animal's mean relationship with the reference data set gave a poor prediction of accuracy (Figure 2a). The best predictor of accuracy was an animal's mean top 10 relationships with the reference (Figure 2b), whereas its highest relationship to the reference was also a good predictor of accuracy (Figure 2d).

## Discussion

The relationship between the animals in the test and reference data sets has an effect on the accuracy of genomic predictions. Close relationships between the two data sets' result in the highest accuracy for GEBV. Similar results were predicted by Hayes et al. [5] and observed by Habier et al. [3,4] for populations that share a close relationship. However, breeding values that are predicted for closely related animals using the traditional pedigree-based BLUP approach also achieve high accuracy. The current study has shown that when there is a distant relationship between the animals in the test and reference data sets, gBLUP is still able to predict an animal's breeding value with some accuracy. Furthermore, when the animals are unrelated by pedigree or when the pedigree relationships are low, gBLUP can use information from distant relatives to maintain a proportion of accuracy of the GEBV.

The information gathered from only distantly related animals enabled an estimate of breeding value to be made with some accuracy. However, when relatives were included in the reference data set, the importance of information on distantly related animals may be reduced. Selection index theory shows that when information on closely related animals is available, more weight is placed on this information and therefore information from distantly related animals becomes less important. Although the importance of information from distant relatives is reduced, this extra information, which is not used in pedigree-based methods, enables gBLUP to achieve a higher accuracy of the EBV. The inclusion of information on relatives improves the accuracy of the predicted breeding values.

If there are no close relationships between animals in the reference and test data sets, the accuracy of the GEBV is driven by distant relationships, which will be more useful when there is more LD in the population. The accuracy obtained for these animals can be called the 'baseline accuracy', which is the accuracy that may be expected for a member of the population that does not have any close relatives in the reference data set. Goddard [6] and Daetwyler et al. [15] proposed predictive formulae for the accuracy of genomic predictions. These methods depend on the size of the reference data set, the effective population size of the breed, the heritability of the trait and the length of the genome [6]. The overall *N_e _*will govern the effective number and size of chromosome segments (*M_e_*) that are segregating in the population. If the effective population size is small, it is expected that animals will share larger chromosome segments and the genomic predictions will be more accurate [5,6]. The accuracy (r) for an individual with no phenotype, as described by Goddard [6], is then predicted as:

$$\text{r}=\sqrt{1-\text{λ}/\left(2\text{N}\text{√}\text{a}\right)\cdot \text{log}\left(\text{ρ}/\text{ρ}\right)}$$

Where ρ = (1 + a+ 2√a), with a = 1+2*λ/*N *and *N *is the number of animals in the reference,

λ = σ^2^_e _/σ^2^_u _where σ^2^_e _is the residual variance and σ^2^_u _is the genetic variance at a single locus and is estimated by σ^2^_u _= h^2^/*M_e _*·k where *M_e _*= 2*N_e_L *and is the effective number of chromosome segments, h^2 ^is the heritability and k = 1/log(2*N_e_*). For the simulation example *N *= 1750, *N_e _*= 100, h^2 ^= 0.3 and *L *= 30. Then k = 0.189, λ = 3773.8, a = 5.31 and consequently the accuracy for an individual with no phenotype was equal to 0.36. Similarly, the alternative method described by Daetwyler et al. [15] results in a predicted accuracy of 0.28 (details not shown). The predicted accuracies resulting from either method were similar to the baseline accuracy in our study achieved by gBLUP in unrelated individuals (0.34). In the theoretical prediction methods, there is some ambiguity about the approximation of *M_e _*[5,23], with proposed values equal to: a) 2*N_e_L/ln(*4*N_e_L)*; b) 4*N_e_L *and c) 2*N_e_L*. Using [6] for each of these values results in predicted accuracies of a) 0.74 b) 0.27 and c) 0.36. Consequently 2*N_e_L *appears to be the most appropriate variable for baseline accuracy in our simulation example. For the Merino sheep data, with an estimated *N_e _*of approximately 1,000 [24], the expected accuracy was 0.15 and lower than that achieved by gBLUP for EMD (0.28) and for SC_WT (0.18). This increase for gBLUP in the real data is possibly due to extra information from animals that shared a genomic relationship but were unknown in the pedigree, or the estimation of *N_e _*may have been affected by heterogeneity of the breed, which really consists of several sub-populations.

Accuracy estimated using the prediction error variance of the mixed model equations (*r*_(*PEV*)_) was shown to be a good approximation of empirical accuracy for the simulation example. Estimated and empirical accuracies were also very similar when using gBLUP for the EMD example. However, some differences between *r*_(*PEV*) _and empirical accuracy were observed for both, BLUP-D and gBLUP in real data in the case of SC_WT. In the simulation example, the empirical accuracy was the correlation between the TBV and EBV (or GEBV), whereas in the Merino data example, the empirical accuracy was the correlation between the ASBV and EBV (or GEBV). The ASBVs are progeny test estimates and have some prediction error associated with them. The empirical accuracy was also likely to be affected by sampling because of the small size of each test data set (50-60 animals). Furthermore, unlike the simulation data, where all animals were linked by a true pedigree, many Merino animals in the unrelated test set had no direct pedigree relationships with the reference data set and therefore only zero breeding values were estimated for these animals. In contrast, in the case of missing pedigree, gBLUP could use genomic relationship information and a more accurate breeding value was estimated for all animals in the test set.

Another complexity in our real data example is the heterogeneity of the Merino sheep population, as it consists of many sub-populations. In routine ASBV analyses, this population structure is accounted for using pedigree information and genetic groups based on individual flock data. When correlating GEBV and ASBV, we accounted for sub-population effects by assigning sires to groups of "fine wool", "medium wool" and "strong wool". Empirical accuracies for SC_WT were clearly affected by correcting for the sub-population structure, which may explain why there are some differences between r_*(*PEV*) *_and r_(cor) _for this trait. The corrections had little to no effect on empirical accuracy for EMD. Note that EMD was corrected for SC_WT and this may have removed some of the sub-population effects on EMD.

The makeup of reference data sets is an important factor for the design of genomic evaluation systems to enable additional genetic gain from genomic selection at the lowest cost. This is especially true for beef cattle and sheep breeding programs that do not have a distinct nucleus tier. We have shown that genomic predictions are more accurate when animals are related to the reference data set; however substantial baseline accuracy can be achieved for all animals in the population. To achieve this, the reference data set will need to include a large number of animals that cover the genetic diversity of the given population (breed). It may be important to include animals that are expected to contribute more to the future gene pool in that breed but these contributions need to be balanced by contributions to genetic diversity [8].

The optimal size of the reference data set will depend on *N_e _*of the given population; populations with higher *N_e _*may need a larger reference data set so that suitable baseline accuracies can be achieved. If the baseline accuracy is low (large *N_e _*and small reference data set size) the contribution of relatives' information will be larger, however this information from relatives is only limited to closely related individuals and will not last over many generations.

## Conclusions

The relationship between animals in the reference and test data sets affects the accuracy of predicting breeding values using gBLUP. When there is a close relationship between the animals in the reference and test data sets, gBLUP can estimate breeding values with a high accuracy. When there is only a distant relationship between the animals in test and reference data sets, gBLUP can still estimate a breeding value with some accuracy. This baseline accuracy depends on the effective population size and the size of the reference data set, and should be carefully considered when designing a reference data set for a breeding program.

## Competing interests

The authors declare that they have no competing interests.

## Authors' contributions

SAC performed the simulation, analyses and drafted the manuscript. JHJW, JMH, HDD and SAC conceived and designed the experiment. All authors have read and approved the final manuscript.

## Appendix 1: Accuracy estimated using the PEV of the mixed model equations weighted by genomic relationships

Firstly the variance of a is defined as:

$$\text{var}\left(\text{a}\right)=\sigma_{\text{a}}^{2}.G_{ii}$$

where $\sigma_{a}^{2}$ is the additive genetic variance and G_ii _is the diagonal of the numerator relationship matrix (or genomic relationship matrix). The prediction error variance is defined as:

$$\text{PEV}=\text{var}\left(\text{a}-\hat{\text{a}}\right)=\sigma_{e}^{2}C_{ii}$$

where $\sigma_{e}^{2}$ is the residual variance and **C***_ii _*is the diagonal of the inverse of the coefficient matrix, furthermore:

$\text{var}\left(\text{a}-\hat{\text{a}}\right)=\text{var}\;\left(\text{a}\right)-\text{var}\;\left(\hat{\text{a}}\right)$ where var($\hat{\text{a}}$) is the estimate of the variance of a and is equal to:

$$\text{var}\;\left(\hat{\text{a}}\right)=\text{var}\;\left(\text{a}\right)-\left(1-r^{2}\right)\text{var}\left(\text{a}\right)$$

Therefore the regression coefficient (r^2^) is equal to:

$${\text{r}}^{2}\text{ = var(a) - var}\left(\text{a}-\hat{\text{a}}\right)/\text{var}\;\left(\text{a}\right)$$

recall: $\text{var}\left(\text{a}\right)=\sigma_{\text{a}}^{2}G_{ii}$ and

$$\text{PEV}=\text{var}\left(\text{a}-\hat{\text{a}}\right)=\sigma_{e}^{2}C_{ii}$$

Therefore:

$$\begin{array}{c} {\text{r}}^{2}=\sigma_{\text{a}}^{2}.G_{ii}-{\text{V}}_{\text{e}}C_{ii}/\left(\sigma_{\text{a}}^{2}G_{ii}\right)\\ \;=\text{1 - }{\text{V}}_{\text{e}}C_{ii}/\left(\sigma_{\text{a}}^{2}G_{ii}\right) \end{array}$$

Finally the accuracy (r) of the estimated breeding value is then given by:

$$\text{r}=\sqrt{1-\sigma_{e}^{2}C_{ii}/\left(\sigma_{a}^{2}.G_{ii}\right)}$$
